# P-259. Dyslipidemia and Its Relationship with Hypertension and Diabetes in HIV Patients: A Cross-Sectional Study in the Dominican Republic

**DOI:** 10.1093/ofid/ofaf695.480

**Published:** 2026-01-11

**Authors:** Yori A Roque, Valerin Carrasco, Angel Lopez, Yoel Garcia, David De Luna

**Affiliations:** Hospital Metropolitano de Santiago HOMS, Santiago, Santiago, Dominican Republic; Hospital Regional Universitario Presidente Estrella Ureña, Santiago de los Caballeros, Santiago, Dominican Republic; Hospital Regional Universitario Presidente Estrella Ureña, Santiago de los Caballeros, Santiago, Dominican Republic; Clínica Universitaria Unión Médica, Santiago de los Caballeros, Santiago, Dominican Republic; Hospital Metropolitano de Santiago, Santiago, Santiago, Dominican Republic

## Abstract

**Background:**

Advances in the management of HIV and AIDS have shifted the primary health concerns for these patients from opportunistic infections to cardiovascular diseases. Although antiretroviral therapy (ART) has significantly improved survival, it has also been linked to complications such as dyslipidemia, hypertension, carbohydrate metabolism disorders, and coronary and peripheral artery disease. In the Dominican Republic, a middle-high-income country, healthcare practices for people living with HIV are generally not focused on preventing these comorbidities.

**Table 1.**
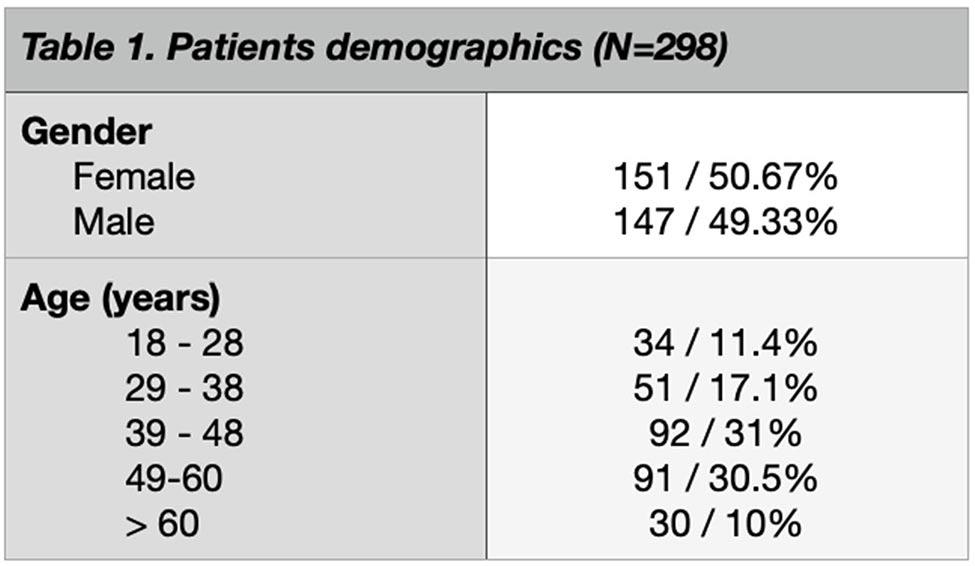
Patients demographics

**Figure:**
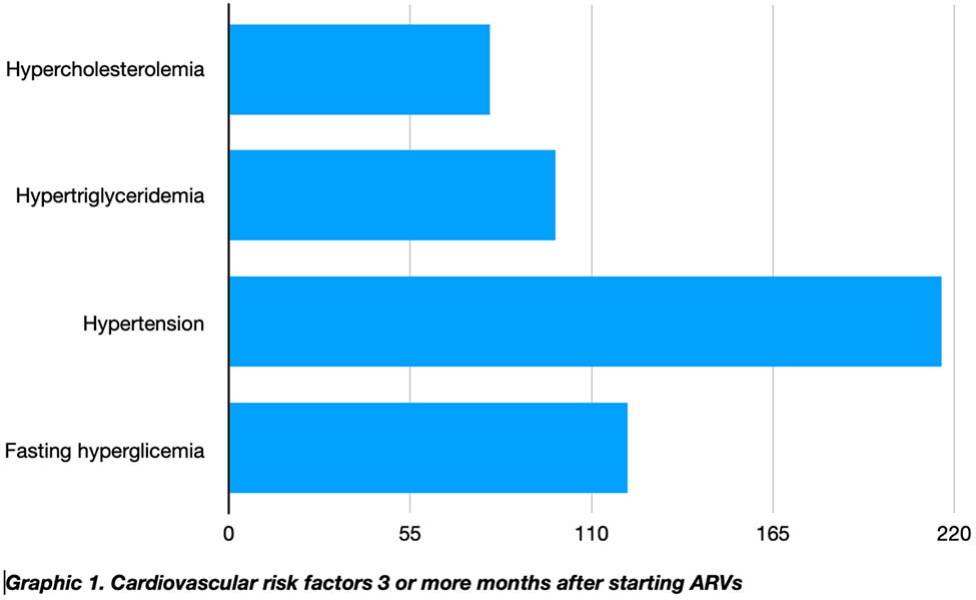
Cardiovascular risk factors 3 or more months after starting ARVs

**Methods:**

This cross-sectional study analyzed data from the HIV care program at a tertiary hospital in Santiago de los Caballeros, Dominican Republic. The aim was to identify and characterize dyslipidemia and its relationship with hypertension and diabetes mellitus. Lipid profiles, serum glucose levels, and blood pressure readings were compared before and at least three months after starting antiretroviral treatment to assess any significant changes.

**Figure:**
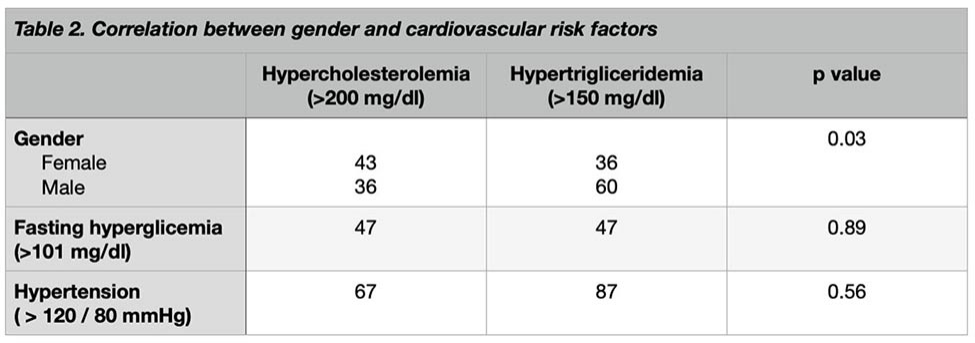
Correlation between gender and cardiovascular risk factors

**Results:**

A total of 298 medical records were reviewed, with 82% of patients on an antiretroviral regimen consisting of Tenofovir/lamivudine + Dolutegravir. Hypertriglyceridemia was the most common form of dyslipidemia (33.2%), followed by hypercholesterolemia (26.5%). Hypertension was prevalent in 72.5% of patients, and 40.6% exhibited fasting hyperglycemia. Dyslipidemia was significantly associated with gender (p=0.03), but no statistically significant relationships were found between dyslipidemia, hypertension, and hyperglycemia.

**Conclusion:**

The high prevalence of hypertension and diabetes or pre-diabetes in the studied population highlights the need for preventive measures to address these chronic non-communicable diseases in HIV patients. It is crucial for healthcare providers to implement lifestyle modifications and consider medications that could slow the development of these complications.

**Disclosures:**

All Authors: No reported disclosures

